# Advantages of human opsins in optogenetic visual restoration

**DOI:** 10.3389/fnins.2025.1725264

**Published:** 2025-11-28

**Authors:** Mihai Teodor Bica, Jasmina Cehajic-Kapetanovic

**Affiliations:** 1 Oxford University Hospitals NHS Foundation Trust, Oxford, United Kingdom; 2 Nuffield Laboratory of Ophthalmology, Nuffield Department of Clinical Neurosciences, University of Oxford, Oxford, United Kingdom

**Keywords:** inherited retinal degenerations, RGCs, BP cells, optogenetic therapy, microbial opsins, mammalian opsins, human-based opsins, channelrhodopsins

## Abstract

Optogenetic vision restoration has progressed from proof-of-concept to early clinical testing, yet most programmes rely on microbial channels that demand high irradiance and offer limited adaptation. This review synthesizes preclinical and clinical evidence comparing microbial actuators with human opsins (rhodopsin, cone opsins, melanopsin) and outlines vector and safety considerations for translation. Human opsins activate G-protein–coupled cascades, providing intrinsic signal amplification and operation at room-light levels (∼10^11^–10^12^ photons⋅cm^–2^⋅s^–1^), in contrast to the ≥1015 photons⋅cm^–2^⋅s^–1^ typically needed for channelrhodopsins. Rhodopsin and MW cone opsin preserve photopic-range sensitivity (rhodopsin > cone opsin) while delivering millisecond-scale kinetics and adaptation across backgrounds, enabling patterned retinal responses without optical intensification devices; clinical validation without external intensification is pending. Such mammalian pigments also confer bleaching-based light adaptation, whereas microbial tools are photocyclic and can desensitize under steady illumination, limiting sustained contrast encoding. Bistable melanopsin enables durable irradiance coding but with slow dynamics; chimeric designs (e.g., melanopsin–mGluR6, Gloeobacter–human rhodopsin) aim to combine amplification with favorable reset properties. In contrast to human opsins, microbial channels warrant safety considerations including light-dose budgeting (particularly at short wavelengths), potential cytotoxicity from proton or calcium loads, and vector-related ocular inflammation; red-shifted actuators improve photochemical safety margins. Targeting opsins to ON bipolar (ON-BP) cells retains inner-retinal computations (center–surround, ON/OFF segregation, temporal filtering). Engineered adeno-associated virus (AAV) capsids (e.g., AAV2-7m8 intravitreally; AAV8.BP2 subretinally) paired with GRM6 or L7 promoters achieve broad ON-BP expression in rodents but a much more limited expression profile in non-human primates. First clinical studies report acceptable early ocular safety with emerging efficacy signals. We propose accelerating phase I safety human trials of human-opsin vectors with prospectively defined light-exposure budgets and low vision functional endpoints such as navigation, face and object recognition, temporal contrast sensitivity, alongside work on chromophore support, cascade integrity in late degeneration, and scalable vector–promoter solutions. Pharmacological noise suppression in degenerating retinas (e.g., gap-junction blockers or retinoic-acid pathway modulators) may further enhance signal-to-noise without altering opsin biochemistry. Together, these steps can move human-opsin optogenetics from experimental promise to clinically meaningful restoration of light sensitivity.

## 1 Introduction

Inherited retinal degenerations (IRDs) are characterized by progressive loss of rod and cone photoreceptors, with varying survival and remodeling of the inner retina. Even in advanced disease, retinal ganglion cells (RGCs) and bipolar (BP) cells often persist but exhibit circuit rewiring, ectopic synapses and glial sealing that can alter signal routing and gain control (Jones et al., 2003; Marc et al., 2003). Optogenetic therapy seeks to reintroduce photosensitivity into these surviving neurones by ectopic expression of light-sensitive proteins, offering a mutation-agnostic strategy that is not constrained by the genetic heterogeneity of IRDs.

Two broad actuator classes have dominated development, microbial and mammalian or human-based opsins. Microbial opsins (e.g., channelrhodopsins, halorhodopsins) are light-gated ion channels or pumps with millisecond kinetics and straightforward coupling to membrane potential, but they typically require high irradiance for reliable spiking, show limited intrinsic light adaptation, and can desensitize under steady illumination due to photocycle inactivation (Bamann et al., 2008; Gunaydin et al., 2010; Hegemann et al., 2005; Klapoetke et al., 2014). Red-shifted variants (ReaChR, ChrimsonR) mitigate short-wavelength photochemical risk and improve tissue penetration, though still generally demand brighter light than native phototransduction (Klapoetke et al., 2014; Lin et al., 2013; Sengupta et al., 2016). Prolonged activation can also perturb ionic homeostasis and elevate calcium loads, with potential safety implications in chronic use (Ermak and Davies, 2002; Feldbauer et al., 2009).

By contrast, mammalian opsins (rhodopsin, cone opsins, melanopsin) are G-protein–coupled receptors (GPCRs) that engage intracellular amplification, enabling operation at room-light levels and broadening dynamic range (Berry et al., 2019; Cehajic-Kapetanovic et al., 2015; Gaub et al., 2015). Human medium-wavelength (MW) cone opsin and rhodopsin provide fast kinetics and adaptation across backgrounds suitable for patterned vision; melanopsin is bistable and robust for irradiance coding but comparatively slow (Berry et al., 2019; De Silva et al., 2017; Lin et al., 2008; Lucas et al., 2014; Mure et al., 2016). Cell-tailored chimeras such as Opto-mGluR6 (melanopsin–mGluR6) aim to couple non-bleaching photochemistry to the native ON-BP cascade, while newer designs (e.g., Gloeobacter Human Chimeric Rhodopsin - GHCR) seek GPCR amplification with improved photostability (Katada et al., 2023; van Wyk et al., 2015). Comparative analyses increasingly highlight the potential advantages of mammalian tools for sensitivity and adaptive range under clinically realistic illumination (Gilhooley et al., 2022).

Target selection and delivery are central to translational success. RGC targeting via intravitreal adeno-associated viruses (AAV) is surgically simple but bypasses inner-retinal processing (Bi et al., 2006; Lin et al., 2008) and leads to toxic inflammation (Dalkara et al., 2009). Targeting ON-BP cells retains center–surround organization, ON/OFF segregation and temporal filtering, better recapitulating retinal computations (Lagali et al., 2008; Macé et al., 2015). Engineered capsids paired with GRM6 or L7/PCP2 promoters achieve broad opsin expression in ON-BP cells in rodents, overcoming inner limiting membrane barriers that limit conventional intravitreal AAV2 (Cronin et al., 2014; Macé et al., 2015; McClements et al., 2021). ON-BP expression of human opsins (rhodopsin, hOPN4, MW cone) has restored retinal and cortical responses and improved behavioral readouts without detectable adverse effects on ON-BP survival in preclinical studies. However, retinal expression profile remains limited in non-human primate (NHP) model which most closely recapitulates human eye (Seitz et al., 2017; Yin et al., 2011) and since no retinitis pigmentosa NHP model exists, it is difficult to determine functional outcomes and vision restoration potential.

Clinical translation is underway. Early studies with microbial actuators have reported acceptable ocular safety and emerging functional signals such as partial visual behaviors with ChrimsonR-based therapy and letter-score gains with multi-characteristic opsins, yet also highlight the need for higher sensitivity at safe light levels and rigorous inflammation control (Boyer et al., 2023; Lam et al., 2025; Sahel et al., 2021). Additional first-in-human experience with a channelrhodopsin construct (RST-001) likewise suggests manageable, low-grade inflammation (AbbViNational Library of Medicine (US)e, 2024). Against this backdrop, the field is poised to evaluate human-opsin vectors that may reduce light-dose requirements, improve dynamic range and leverage retinal adaptation to deliver more naturalistic vision.

Recent surveys summarize optogenetic vision-restoration strategies across microbial, mammalian and chimeric opsins, delivery routes, and early clinical programs (Busskamp et al., 2024; Lindner et al., 2022; Parnami and Bhattacharyya, 2023; Stefanov and Flannery, 2023), with disease-focused perspectives emerging for geographic atrophy (Borchert et al., 2024). Distinct from these, our review concentrates specifically on human GPCR opsins and their translational advantages (biochemical amplification, ambient-light compatibility, and immunologic familiarity), and it adds pragmatic, clinic-ready guidance absent from prior reviews: OCT-based inclusion/exclusion thresholds as a proxy for bipolar-cell survival, quantitative operating ranges to expect in clinic (illuminance/temporal bandwidth), and a mapping from preclinical readouts to patient-centered endpoints (full-field stimulus threshold, microperimetry, performance-based tasks). This focus is intended to help trialists move from mechanism to patient selection and outcome design rather than to recapitulate broad tool catalog.

In this review we (i) compare microbial and mammalian actuators with emphasis on sensitivity, dynamic range and kinetics; (ii) examine light adaptation, bleaching and photocycle behavior; (iii) summarize vector engineering and ON-BP targeting strategies; (iv) assess safety and toxicology considerations including light-dose budgeting; and (v) appraise current clinical data and translational priorities. Throughout, we evidence that human opsins present compelling advantages for real-world vision restoration and outline the key experiments and trial designs needed to realize that potential.

## 2 Methods

This focused review synthesizes preclinical and early clinical work on optogenetic vision restoration that compares mammalian opsins (rhodopsin, cone opsins, melanopsin) with microbial actuators and/or targets ON-bipolar cells. We prioritized *in vivo* mammalian studies reporting retinal photon fluxes (photons⋅cm^–2^⋅s^–1^), dose–response or behavioral readouts, alongside peer-reviewed clinical reports. Literature was identified from PubMed/Google Scholar and reference lists (2003–September 2025) using terms including “optogenetic,” “retinal degeneration,” “human opsin,” “rhodopsin,” “cone opsin,” “melanopsin,” “Opto-mGluR6,” “GHCR,” “channelrhodopsin,” “ON bipolar,” and “AAV.” Selection emphasized translational relevance to room-light operation, safety budgeting and inner-retina targeting.

### 2.1 Sensitivity, dynamic range, kinetics

**FIGURE 1:**
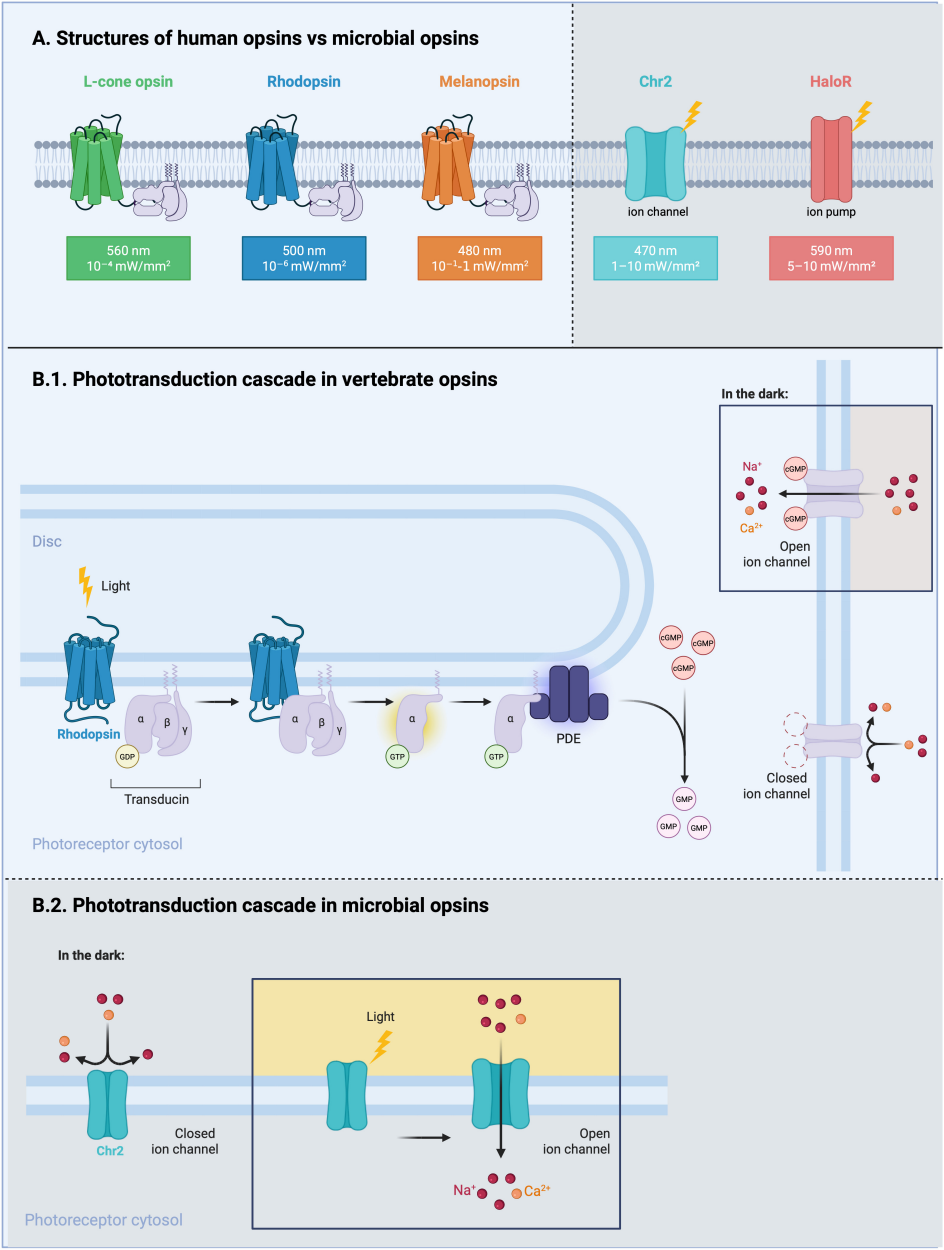
Structural and signaling differences between vertebrate and microbial opsins. **(A)** Structures of human opsins vs. microbial opsins. Transmembrane topologies of three human GPCR opsins: L-cone opsin (λ_*max*_ ≈ 560 nm), rhodopsin (500 nm) and melanopsin (480 nm) are contrasted with the microbial channelrhodopsin-2 (ChR2, 470 nm) and halorhodopsin (HaloR, 590 nm). Approximate irradiances required for activation are indicated beneath each pigment. ChR2 functions as a light-gated cation channel, whereas HaloR operates as a light-driven ion pump. **(B.1)** Phototransduction cascade in vertebrate opsins. Photon capture by rhodopsin activates transducin, which stimulates phosphodiesterase-6, hydrolyzing cyclic guanosine monophosphate (cGMP). The ensuing cGMP decline closes plasma-membrane cyclic-nucleotide–gated Na^+^/Ca^2+^ channels, generating hyperpolarization. In darkness, elevated cGMP maintains these channels in an open state. **(B.2)** Phototransduction cascade in microbial opsins. Illumination directly opens ChR2, permitting Na^+^ and Ca^2+^ influx without second-messenger amplification. Thus, microbial opsins couple light absorption to ionic current in a unimolecular process, unlike the multistep vertebrate cascade. Created in BioRender. Bica, M. (2025) https://BioRender.com/bo0sd62.

A hallmark of rod and cone opsins is their ability to trigger intracellular enzymatic cascades upon photon absorption, greatly amplifying the signal (Sakai et al., 2022; Figure 1). Microbial opsins are light-gated ion channels that lack signal amplification, each absorbed photon directly gates a single channel, yielding a limited current per molecule, unlike mammalian opsins (rod and cone opsin, melanopsin) which activate a G-protein coupled cascade, allowing for intrinsic signal amplification (Bi et al., 2006). Mammalian rhodopsin has exquisite light sensitivity with ability to detect a single photon of light. When fully dark adapted, humans can detect as few as 7–10 photons. The absorption of a single photon is sufficient to alter the membrane conductance through a cascade of amplification steps. A single photoactivated opsin activates tens of G proteins, each of which activates one effector, yielding thousands of second-messenger molecules modified. Classic amphibian estimates often quoted 500 transducin molecules per activated rhodopsin reflecting longer activated rhodopsin lifetimes at room temperature (Burns and Arshavsky, 2005). In mammalian rods, photoactivated rhodopsin is active for ∼40–50 ms and activates transducin at ∼3 × 10^–2^–1.3 × 10^3^ molecules⋅s^–1^, i.e., ∼10–60 transducins per photon depending on preparation and temperature (Arshavsky and Burns, 2012; Chen et al., 2012; Heck and Hofmann, 2001). Each activated transducin then activates a PDE6 holoenzyme whose turnover is ∼3 × 10^3^–5.6 × 10^3^ cyclic guanosine monophosphate (cGMP)⋅s^–1^, so the integrated output per photon is on the order of 10^4^–10^5^ cGMP molecules hydrolyzed, closing ∼3%–5% of cyclic nucleotide-gated (CNG) channels and producing a ∼1 pA single-photon current in amphibian rods with a comparable fractional closure in mammals (Cote et al., 2021; Reingruber et al., 2015; Yau and Hardie, 2009). Representative microbial and mammalian opsins, together with their wavelength, mechanisms and key practical advantages and disadvantages, are summarised in Table 1.

**TABLE 1:** Summary of different opsins used in optogenetic research.

Opsin	Mechanism	λmax	Key characteristics	Advantages	Disadvantages	Original research
ChR2 (channelrhodopsin-2)	Depolarising cation channel	470 nm	∙ Fast ON/OFF (∼1–10/10–20 ms) ∙ High irradiance to drive spikes ∙ Small gene fits AAV	∙ Predictable kinetics ∙ Precise temporal control ∙ Broad toolchain	∙ High light demand ∙ Blue-light photochemical risk ∙ Shallow tissue penetration at blue	Bi et al., 2006; Boyden et al., 2005; Lagali et al., 2008; Nagel et al., 2003
CatCh	Depolarising cation channel (Ca^2+^-permeable mutant)	470 nm	∙ Higher operational sensitivity vs. wt ChR2 ∙ Blue-activated	∙ Lower required irradiance vs. ChR2 for spiking ∙ Can improve spike fidelity at lower light.	∙ Blue-light safety considerations ∙ Increased Ca^2+^ influx may raise excitotoxicity	Kleinlogel et al., 2011
ReaChR	Depolarising cation channel (red-shifted)	590 nm	∙ Amber-red activation ∙ Moderate kinetics (tens of ms) ∙ Improved tissue penetration vs. blue	∙ Reduced blue-light hazard ∙ Better penetration ∙ Useful when longer wavelengths are preferred	∙ Slower than fastest blue opsins ∙ Still requires relatively bright light for robust responses.	Lin et al., 2013
ChrimsonR	Depolarising cation channel (red-shifted)	590 nm	∙ Trafficking-optimized red actuator ∙ Moderate kinetics ∙ Validated in translational retina contexts	∙ Amber/red drive with improved safety/penetration ∙ Clinical case report support (with goggles).	∙ Requires high-intensity amber stimulation (often with device) ∙ Slower than blue opsins.	Klapoetke et al., 2014; Sahel et al., 2021
Halorhodopsin (NpHR/eNpHR3.0)	Inhibitory Cl pump (hyperpolarising)	590 nm	∙ Light-driven Cl influx ∙ Strong inhibition ∙ eNpHR3.0 improves trafficking/expression.	∙ Robust silencing ∙ Complements depolarising tools for push–pull ON/OFF strategies.	∙ High light demand ∙ Potential Cl homeostasis issues ∙ Pumps are slower and non-amplifying. ∙ Limited use in restoration clinical pipelines	Gradinaru et al., 2010; Han and Boyden, 2007; Zhang et al., 2009
Rod opsin (RHO)	Mammalian GPCR (Gi/o/t cascade)	498 nm	∙ GPCR amplification ∙ Highest sensitivity ∙ Adaptation over wide light range	∙ Self-protein → lower immunogenicity risk; ∙ Indoor-light operation (∼10 –10) ∙ Lower irradiance than microbial channels improves safety margin ∙ ON-BP targeting leverages inner-retina processing ∙ Built-in deactivation via GRK/arrestin	∙ Requires retinal recycling ∙ Slower temporal response may limit image clarity.	Cehajic-Kapetanovic et al., 2015
Cone opsin (MW)	Mammalian GPCR	530 nm	∙ GPCR amplification ∙ High sensitivity ∙ Adaptation over wide light range	∙ Self-protein ∙ Photopic-range operation ∙ Lower irradiance than microbial channels improves safety margin	∙ Requires retinal recycling ∙ Sensitivity lower than rod opsin at the same expression level ∙ Reduced coupling to secondary messengers compared to rhodopsin	Berry et al., 2019
Melanopsin (OPN4)	Mammalian GPCR (bistable)	480 nm	∙ Bistable ∙ Second-scale kinetics; ∙ Irradiance/brightness coding ∙ Sustained responses.	∙ Self-protein ∙ Reduced dependence on RPE visual cycle ∙ Tolerates bright light	∙ Poor temporal resolution for fine image-forming tasks	De Silva et al., 2017; Lin et al., 2008
GHCR (Gloeobacter–Human Chimeric Rhodopsin)	Chimeric rhodopsin (microbial–human GPCR loops)	520 nm	∙ Monostable chimeric design ∙ Dim-light restoration; ∙ Neuroprotection reported in models.	∙ High sensitivity ∙ No exogenous chromophore needed (mouse)	∙ Microbial components ∙ Design-specific tuning required ∙ Chimeric structure with potential expression limits	Katada et al., 2023
Opto-mGluR6	Chimeric GPCR (melanopsin–mGluR6)	490 nm	∙ ON-BP targeting ∙ GPCR amplification; ∙ Daylight-level retinal/cortical/behavioral rescue	∙ Strong sensitivity with inner-retina processing preserved	∙ Construct-specific optimisation ∙ Potential desensitization ∙ Vector and promoter constraints	van Wyk et al., 2015

Early proof-of-principle showed that by expressing mouse melanopsin in RGCs of rd1 mice, there is restoration of pupillary light reflexes and modest visually guided behavior (Lin et al., 2008). Subsequent work extended this approach to the human ortholog, demonstrating that human melanopsin (hOPN4) expression similarly evokes light responses in mice models of advanced rod-cone degenerations (De Silva et al., 2017; Lin et al., 2008). Such G-protein-mediated amplification broadens the dynamic range and sensitivity under moderate indoor lighting: a potential clinical advantage over microbial opsins, which often require very bright stimulation or optical intensification goggles (Berry et al., 2019).

In retinal RGCs expressing Channelrhodopsin-2 (ChR2), light sensitivity is at least four orders of magnitude lower than that of native photoreceptors (even cones). Under matched retinal conditions, unmodified ChR2 typically requires ∼10^14^–10^15^ photons⋅cm^–2^⋅s^–1^ to drive robust spiking, whereas rod-opsin or Opto-mGluR6 strategies elicit responses around ∼10^11^–10^12^ photons⋅cm^–2^⋅s^–1^, corresponding to dim-to-moderate indoor light (Cehajic-Kapetanovic et al., 2015; Pan et al., 2014; van Wyk et al., 2015). Note that reported thresholds depend strongly on illumination geometry and spectrum (full-field vs. spot, LCD vs. LED, spectral match to the opsin), ocular state (pupil size, media absorption), and whether values are given as corneal lux or retinal photons⋅cm^–2^⋅s^–1^, hence, cross-study numbers are most reliable when methods are matched (Figure 1). It is also worth noting that photochemical hazard rises steeply at short wavelengths; action-spectrum data and translational studies support preferential use of longer wavelengths where feasible (Lin et al., 2013; Sengupta et al., 2016; van Norren and Gorgels, 2011).

Studies specifically investigating human rhodopsin in rd1 mice models of retinal degeneration, showed that this provides an increased sensitivity compared to channelrhodopsin-based tools. Whereas ChR2 often demands intensities akin to full daylight, in human rod-opsin–treated rd1 mice, robust retinal and thalamic responses occur at ∼8 × 10^11^–10^12^ rod-equivalent photons⋅cm^–2^⋅s^–1^, with behavioral effects at 20–40 lux on standard LCDs (Cehajic-Kapetanovic et al., 2015). This enhanced photosensitivity is directly attributed to intracellular signal amplification by G-proteins (van Wyk et al., 2015). Cone opsins likewise exhibit photopic-range sensitivity, but require somewhat brighter intensities than rods. The human cone opsin can likewise exploit signal amplification and function in moderate photopic conditions, as suggested in the context of synthetic cone-based optogenetic therapy. MW cone opsin can still function under typical office lighting without the external optical “boost” often needed by microbial opsins and thus delivers ms-scale rise/decay whilst maintaining sensitivity comparable to rhodopsin (Berry et al., 2019). Importantly, ectopic rhodopsin in non-photoreceptor cells is operational over at least 5 log units of light *in vitro* assays (Eleftheriou et al., 2017) and over 3 log units *in vivo* thalamic responses, including detection of contrast with light adapted responses (Cehajic-Kapetanovic et al., 2015). This has not been shown with microbial-based opsins. Collectively, these data reflect the expected trade-off: microbial channels offer speed but demand higher photon fluxes, whereas mammalian opsins leverage amplification to operate at lower light levels.

While pupillary light reflex restoration provides a convenient early marker of visual pathway function, several groups have probed more nuanced behavioral and electrophysiological endpoints. It was demonstrated that human rod opsin-treated rd1 mice navigated a visually guided Y-maze more effectively, achieving a 70% correct choice rate after 6 days of training, whereas untreated rd1 controls failed to reach this threshold (Gaub et al., 2015). These absolute improvements establish that mammalian opsin-based therapies can restore meaningful functional vision beyond rudimentary pupil responses in models of advanced retinal degeneration. Broader suites of behavioral assays were designed to capture multiple facets of functional vision in more naturalistic contexts. Going beyond simple light/dark transitions, an open-field paradigm (similar to a light/dark box) was employed, in which mice could move freely between two arenas while experiencing changes in illumination, flickering screens, and coarse spatial patterns projected on LCD monitors (Cehajic-Kapetanovic et al., 2015). The uniqueness of this approach is its reliance on spontaneous locomotor responses to abrupt light increments, moderate-contrast drifting gratings, and flicker at various frequencies. The recorded change in behaviors indicates that treated mice can detect changes under light levels comparable to typical indoor environments. Even more so, these animals also responded to complex stimuli: when shown a naturalistic video clip of a swooping owl, treated rd1 mice exhibited a clear shift in movement patterns, demonstrating that the ectopic rod opsin not only restored sensitivity to basic luminance and contrast but also allowed the retina to encode dynamic, real-world scenes.

Additionally, subretinal delivery of melanopsin in adult mice demonstrated measurable improvements in light-driven cortical responses- highlighting that mammalian opsin therapies can influence higher-order visual processing (De Silva et al., 2017). These consistent improvements over baseline suggest that human opsins, when correctly targeted (e.g., to BP cells), can impart meaningful functional benefits beyond reflexive behaviors. Expression of a mammalian MW cone opsin in mouse RGCs enabled behavioral discrimination of spatial and temporal patterns on a standard LCD display under room light, with responses elicited by brightness steps as small as ∼25% and with adaptation spanning ∼2–3 log units under room light and did so without intensification goggles (Berry et al., 2019). Overall, the human opsin mediated intrinsic gain via GPCR cascade, amplification, range extension and light adaptation enable operation at lower light levels and a broader, more physiological dynamic range under photopic and scotopic conditions compared to microbial opsins.

While human opsins confer enhanced photosensitivity and lower light requirements through G-protein-mediated signal amplification, it is also important to note that the metabotropic signaling cascade can have slower kinetics than direct ion channel gating. In ON-BP implementations, rhodopsin’s cortical time-to-peak is ∼0.8–1.0 s with decay ∼1.0–1.4 s at modest intensities (Gaub et al., 2015), improving at higher irradiance, reflecting fast BP cell cascade kinetics but potentially reduced levels of “shutoff proteins” in BP cells at late stages of degeneration. In contrast, microbial opsins like ChR2 activate and deactivate rapidly due to their direct ion channel gating, allowing for higher-frequency stimulation, but no potential for amplification and dynamic range extension, making them very light insensitive with limited operational dynamic range. Patch-clamp studies on wild-type ChR2 consistently place its off time-constant in the 9–12 ms range at room temperatures with sub-millisecond activation (0.4–1 ms) (Bamann et al., 2008; Hegemann et al., 2005). These values mean that a single ChR2 molecule can in principle follow light pulses delivered at 100 Hz before appreciable desensitization sets in. Faster engineered variants such as ChETA shorten the off time-constant to a few milliseconds (∼3 ms), supporting high frequency spiking (up to ∼200 Hz) in cortical neurones, albeit at the cost of smaller photocurrents (Gunaydin et al., 2010). Such kinetics are much faster than those elicited by native human opsins, so the quality of visual image perception post optogenetic therapy could be compromised. Other advances in microbial opsin engineering (e.g., modified ChrimsonR variants) seek to improve their sensitivity (Klapoetke et al., 2014), though they still lack the intrinsic adaptation capabilities that come naturally with mammalian opsins. When the same opsins are examined with multi-electrode arrays (MEA) in degenerate mouse retina, the effective temporal resolution is lower, because spikes are driven only after membrane integration and synaptic transmission. In one MEA study of ChR2-positive RGCs, the mean time-to-peak of the light-evoked spike histogram was around 50 ms and reliable following was observed up to 20–40 Hz (Reh et al., 2018). Therefore, the biophysical limit set by channel gating (approximately 10 ms) is not the bottleneck in intact retinal tissue, but rather the circuit and synaptic delays.

For comparison, commercial epiretinal implants such as Argus II deliver biphasic current trains at 3–60 Hz in order to avoid perceptual fading and charge-density limits (Stronks and Dagnelie, 2014). By way of benchmark, the highest visual acuity reported with a retinal prosthesis (Alpha AMS) after intensive rehabilitation is ∼1.39 logMAR (20/500; 6/150) (Cehajic Kapetanovic et al., 2020), with the added need of surgical hardware.

### 2.2 Light adaptation, bleaching, and photocycle behavior

**FIGURE 2:**
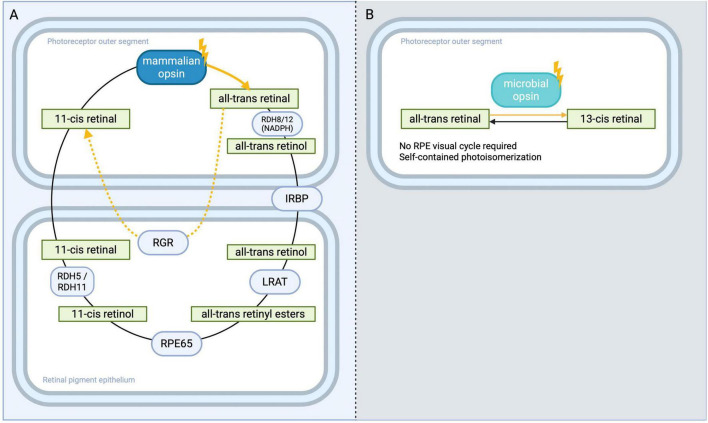
Chromophore regeneration in vertebrate versus microbial opsins. **(A)** Vertebrate visual cycle. Photon absorption (gold arrow) converts 11-cis retinal → all-trans retinal within the mammalian opsin (“bleaching”). The all-trans retinal is rapidly reduced by RDH8/12 (NADPH) to all-trans retinol and trafficked to the RPE via IRBP. In the RPE, LRAT esterifies retinol to all-trans retinyl esters; RPE65 then isomerises–hydrolyses these esters to 11-cis retinol, which RDH5/RDH11 oxidize to 11-cis retinal for return to the outer segment. A light-dependent auxiliary branch mediated by RGR-opsin in the RPE and a subset of Müller glia photoisomerizes all-trans retinal → 11-cis retinoids (gold arrows), augmenting the dark-active, enzyme-driven cycle (solid dark arrows). **(B)** Microbial photocycle. Microbial opsins contain a self-contained chromophore cycle: all-trans → 13-cis retinal upon illumination (gold arrow), followed by thermal relaxation 13-cis → all-trans (solid dark arrow), eliminating any requirement for the RPE visual cycle and supporting rapid, repetitive activation–deactivation. Created in BioRender. Bica, M. (2025) https://BioRender.com/hfvms3j.

Mammalian opsins and microbial opsins differ fundamentally in how they respond to sustained illumination and how they “reset” after photon capture. Vertebrate visual opsins (rod/cone opsins) are bleachable pigments: upon photon absorption, the chromophore (11-cis retinal) is isomerized to all-trans and eventually dissociates, rendering the opsin inactive until the chromophore is enzymatically regenerated (Figure 2). This bleaching leads to an activity decline in continuous light, but it also underlies an important adaptation mechanism. In human cone opsins, for example, persistent bright light causes a progressive reduction in available pigment (bleaching adaptation), effectively desensitizing the cell and preventing over-saturation. Systematic comparisons likewise note improved dynamic-range behavior for mammalian opsins over channelrhodopsins in matched paradigms (Gilhooley et al., 2022). In addition to the dark-active, enzyme-driven RPE65 pathway, mammalian retina also uses a light-dependent (photic) route in which retinal G-protein-coupled receptor (RGR) opsin catalyzes photoisomerization of all-trans-retinal to 11-cis-retinal/retinol in the RPE and specialized Müller glia (Figure 2). Recent mouse genetics and biochemistry show that this RGR pathway accelerates chromophore recycling under sustained light, supporting cone function and even influencing rod dark adaptation; when RGR is disrupted, cone sensitivity and dark adaptation are impaired (Tworak et al., 2023). In our framework we treat the RGR route as a secondary/auxiliary source that augments chromophore supply in bright conditions, whereas the RPE65 pathway remains the primary baseline cycle. The existence of two independent pathways has benefits for optogentic vision restoration in cases where one pathway is more affected then the other by retinal degeneration, then the less affected pathway can still support visual pigment recycling.

When mammalian opsins (rod, cone, or melanopsin) are expressed ectopically, there is potential for similar adaptation. Ectopic rod opsin in a degenerated retina will bleach under high illuminance, making its effective pigment concentration inversely proportional to background irradiance. The positive aspect of this is that light adaptation allows sustained responses over time: as bleaching curtails further activity in bright conditions, it creates room for the neurone to respond to changes (flicker or dimming) on top of the background. In treated mice, human rod opsin enabled detection of modest brightness changes and flicker under light-adapted conditions without saturating (e.g., responses at ∼20–40 lux on LCD screens; retinal ∼10^11^–10^12^ rod-equivalent photons⋅cm^–2^⋅s^–1^; detectable 2–10 Hz flicker; coarse gratings) (Cehajic-Kapetanovic et al., 2015). This mirrors natural retinal behavior where rods saturate in photopic light and cones take over – in therapy, rod opsin can mediate vision in mesopic ranges, while cones or other mechanisms might handle brighter contexts.

Operationally, MW cone opsin exhibits intrinsic light adaptation capabilities, shifting its sensitivity in response to ambient light, thereby normalizing background luminance and extending visual function from indoor to outdoor illumination (Berry et al., 2019). This built-in adaptation allowed MW-opsin-treated mice to successfully perform light-guided tasks across varying lighting conditions. In a comparison of optogenetic tools for vision restoration, mammalian opsins, particularly MW-opsin, were noted to provide superior dynamic range adaptation compared to microbial opsins (Gilhooley et al., 2022).

Microbial opsins, in contrast, are photocyclic pigments that retain their chromophore and recover via thermal or photochemical steps rather than an RPE-dependent visual cycle (Figure 2). Channelrhodopsins and halo-/archaeorhodopsins keep their retinal chromophore bound and can be reactivated repeatedly; after photoexcitation, they transition through intermediate states and eventually return to a ground state, either via spontaneous thermal relaxation or under ambient light. They therefore do not depend on the retinal pigment epithelium for chromophore supply. While this means microbial opsins can, in principle, continuously respond without needing a chemical reset, it comes with trade-offs. Continuous illumination of ChR2 leads to a pronounced desensitization (the photocurrent decays within hundreds of milliseconds even if light remains on), and the channel enters a non-conducting state from which it must recover in the dark (Bamann et al., 2008; Hegemann et al., 2005; Klapoetke et al., 2014). Thus, a cell with microbial opsin under steady light might either fire persistently (if the opsin does not inactivate) or cease firing due to opsin inactivation with neither scenario providing a useful encoding of steady light intensity changes. Additionally, microbial opsins lack the sophisticated calcium feedback and network adaptation that vertebrate photoreceptors use to adjust to background light. Head-to-head comparisons emphasize that cone-opsin implementations adapt across backgrounds whereas ChR2 implementations do not, contributing to the need for optical intensification with microbial channels (Berry et al., 2019; Gilhooley et al., 2022). This limitation contributes to poorer sustained visual responses under varying illumination: e.g., an RGC with ChR2 cannot easily adapt its sensitivity when moving from a dark room to bright sunlight, whereas a BP cell with human rod opsin could adjust via bleaching and downstream network modulation.

Photobleaching does introduce some challenges for human opsin-based therapy. In end-stage retinal degeneration, the normal visual cycle is disrupted due to loss of photoreceptors and RPE (retinal pigment epithelium), so replenishment of 11-cis retinal may be limited (Katada et al., 2023). Without supplementation, bleached opsins may accumulate in an inactive all-trans form. This raises concerns about sustained function: a human opsin therapy might initially restore light responses, but if the opsin repeatedly bleaches and cannot be regenerated, the rescued visual function could wane with continued exposure. In rd1 retina, rod-opsin responses were still elicited at physiological backgrounds, and the authors discuss both residual cis-retinal availability and exogenous supplementation as fall-backs (Cehajic-Kapetanovic et al., 2015).

Melanopsin, although an animal opsin, is atypical in that it is not easily photobleached, it has a bistable photopigment cycle, meaning it can be restored to a ground state by absorbing a second photon of a different wavelength. This property allows melanopsin-expressing cells (such as intrinsically photosensitive RGCs) to sustain responses over long illumination periods without dependence on the RPE (Lucas et al., 2014). Indeed, melanopsin gene therapy in blind mice produced stable behavioral light responses for up to 13 months post-injection (De Silva et al., 2017). The trade-off is that melanopsin’s bistability and intrinsic kinetics result in very slow response dynamics (ON responses lasting seconds) and require distinct light wavelengths for pigment reset (Lucas et al., 2014; Mure et al., 2016). This could lead to an “unnatural” visual experience if a secondary light (usually a longer wavelength) was needed to switch off the pigment.

Chimeric approaches strive to combine advantages and eliminate drawbacks. For instance, Opto-mGluR6 (a fusion of human melanopsin’s light-sensing domain with the ON-BP cell mGluR6 receptor domain) couples a non-bleaching opsin to the native retinal cascade. It avoids photobleaching and drives ON-BP responses with latencies on the order of ∼25–50 ms and behavioral thresholds near 5–6 × 10^13^ photons⋅cm^–2^⋅s^–1^ (van Wyk et al., 2015).

**FIGURE 3:**
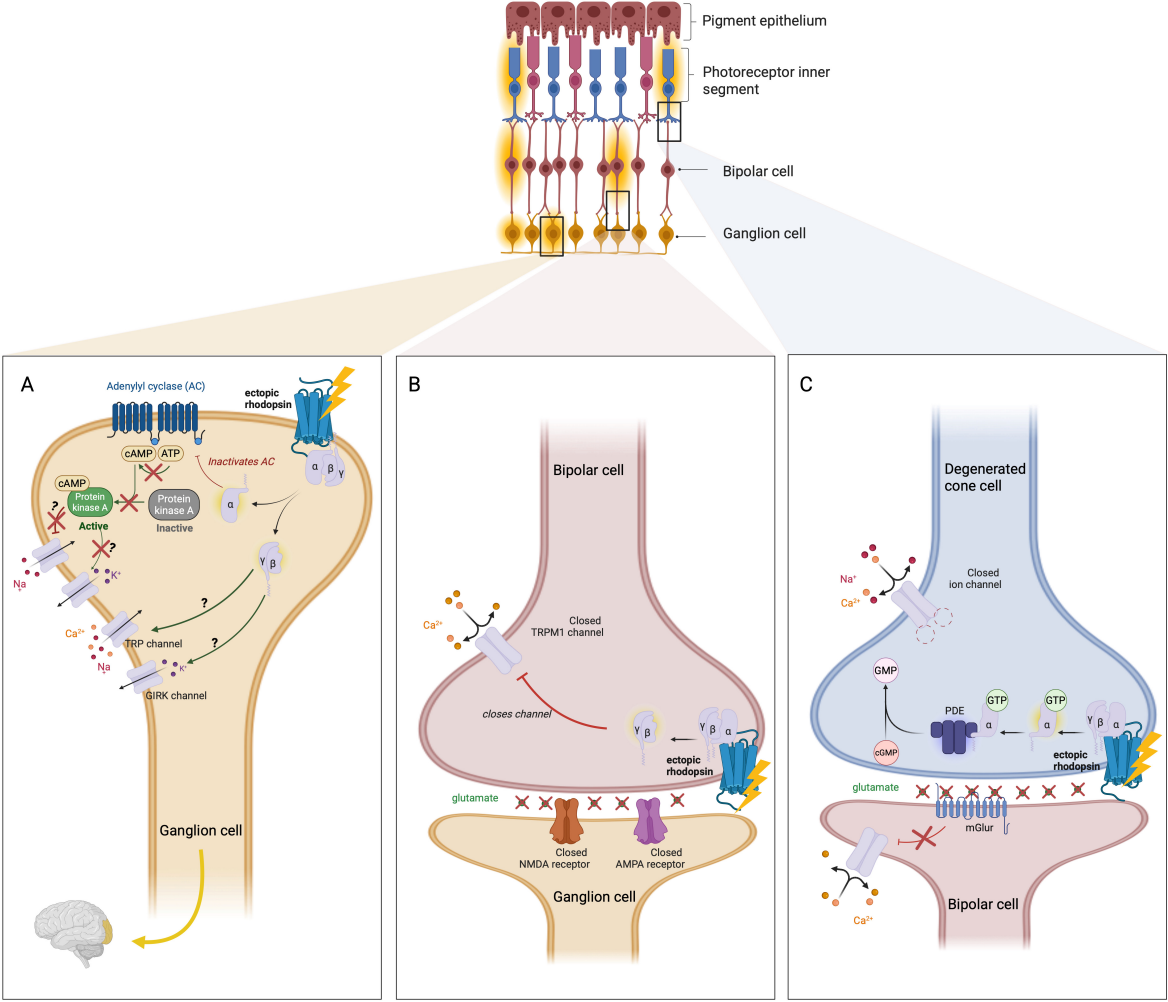
Molecular mechanisms of ectopic rhodopsin-mediated signaling for optogenetic vision restoration. Schematic representation of three contexts in which human rod opsin (rhodopsin) is expressed ectopically in the degenerated retina to restore light sensitivity. Top: Illustration of the retinal structure with expression of ectopic rhodopsin either ubiquitously, in retinal ganglion cells (RGCs), bipolar (BP) cells or in degenerated inner photoreceptor segments. **(A)** RGC expression. Ectopically expressed rhodopsin couples to endogenous Gi/o proteins. Upon light activation, the Gα subunit inhibits adenylyl cyclase (AC), reducing cAMP levels and inactivating protein kinase A (PKA), which may affect downstream ion channels. The freed Gβγ dimer can modulate ion conductances, potentially opening GIRK channels (leading to hyperpolarization) or altering TRP channel activity (which may depolarize the cell). Due to lack of native phototransduction machinery (e.g., PDE6, cGMP-gated channels), the net effect is cell-type-dependent and variable, but in some RGCs results in weak, light-evoked spiking. **(B)** ON-BP cell expression. Ectopic rhodopsin activates the native mGluR6 signaling cascade. Light-driven activation of Gi/o leads to Gβγ-mediated closure of TRPM1 cation channels, mimicking the effect of glutamate binding to mGluR6 in the dark. This results in hyperpolarization of the BP cell and a reduction in glutamate release onto downstream RGCs. Although the polarity of response is inverted compared to the native ON pathway, modulation of glutamate output is preserved. **(C)** Remnant cone photoreceptor expression. Light activation of ectopic rhodopsin triggers the canonical phototransduction cascade. Activated transducin (Gα) stimulates PDE, which hydrolyses cGMP, leading to closure of cGMP-gated cation channels in the outer segment membrane. This causes hyperpolarization and reduced glutamate release onto ON-BP cells. The downstream BP cell expresses mGluR6, which remains inactive in light, allowing TRPM1 channels to open and the cell to depolarize and thus preserving natural signal polarity. Created in BioRender. Bica, M. (2025) https://BioRender.com/hm4f3tn.

Native melanopsin (OPN4) in intrinsically photosensitive RGCs (ipRGCs) shows intrinsically slow phototransduction: dim-flash responses are dominated by two slow steps with τ≈ 2 s and ≈ 20 s, yielding latencies/time-to-peak on the order of seconds, and “OFF” recovery often ∼20–23 s. Additionally temporal bandwidth is low (flicker ≤ 0.2 Hz) (Chen et al., 2012; Schmidt et al., 2008; Walch et al., 2015). In terms of sensitivity, intrinsic ipRGC activation typically requires bright retinal irradiance (thresholds around ∼11 log photons⋅cm^–2^⋅s^–1^, higher than rods or cones), and *ex vivo* recordings commonly use ∼10^13^–10^14^ photons⋅cm^–2^⋅s^–1^ at 480 nm to evoke robust melanopsin currents (Wong et al., 2005). By contrast, Opto-mGluR6 in ON-bipolar cells produces faster kinetics: RGC latency-to-first-spike ∼73 ± 33 ms (vs. ∼84 ± 18 ms with photoreceptors in the same prep), ON-bipolar latency-to-peak ∼25–50 ms (OFF ∼100 ms), with half-maximal responses at ∼1.6–2.3 × 10^–13^ photons⋅cm^–2^⋅s^–1^ (threshold ∼5 × 10^11^; saturation ∼5 × 10^14^) (van Wyk et al., 2015). Mechanistically, this acceleration reflects coupling to the endogenous mGluR6-TRPM1 cascade in ON-bipolar cells rather than the slower Gq-TRP pathway intrinsic to ipRGC (Figure 3).

Another example is the recently developed GHCR (Gloeobacter Human Chimeric Rhodopsin), which merges a microbial rhodopsin backbone with human opsin loops. GHCR was designed to be a monostable pigment like vertebrate rhodopsin (so it activates a G-protein cascade) but without irreversible bleaching. In other words, it aims to achieve high sensitivity and amplification like rod opsin, yet behave more like microbial opsins in stability. Initial results in mice show that GHCR restored light responses in degenerated retina with good sensitivity and no need for external chromophore supply (Katada et al., 2023).

Such innovations highlight that balancing light adaptation and bleaching is critical: human opsins naturally confer beneficial light adaptation and amplification, but their deployment must ensure chromophore availability or use engineered bistable variants.

Despite these positive findings, some studies raise questions about whether exogenous opsins, particularly in severely degenerate retinas, fully integrate into the normal phototransduction machinery. For example, while human opsin therapies leverage the existing G-protein cascade, the extent to which downstream elements (e.g., transducin, phosphodiesterase) remain functional in end-stage disease is not fully understood. This uncertainty is compounded by well-documented retinal remodeling in degeneration (rewiring, ectopic synapses, glial sealing), which can alter cascade components and signal routing (Jones et al., 2003; Marc et al., 2003). Further electrophysiological experiments are needed to establish at what extent ectopically expressed rod or cone opsins truly recapitulate the full bleaching–regeneration cycle and produce stable, repeatable photoresponses in chronically diseased retinal circuits. Addressing such uncertainties will help optimize dosing and vector design for patients at different stages of photoreceptor loss.

Although further psychophysical or behavioral testing is needed to quantify this advantage in human patients, these early *in vivo* data suggest that dynamic reconstitution and bleaching-adaptation in human opsins could facilitate more naturalistic vision restoration in day-to-day environments. Overall, human opsins offer a more “photoreceptor-like” light response, with adaptation and recovery akin to normal vision, compared to the more rigid response of microbial channels.

### 2.3 Vector engineering and targeting

Perhaps equally important to the choice of opsin is the choice of neuronal cell type that receives the optogene. Some early optogenetic therapies focused on RGCs because of the relative ease by which they can be transduced using intravitreal injections (Figure 3; Bi et al., 2006; Lin et al., 2008). This approach however bypasses the retina’s natural circuitry and fails to harness its potential for specialized computations.

Targeting opsin expression to BP cells is highly advantageous because it retains the intrinsic signal processing of the inner retina (Figure 3). BP cells occupy the normal position of photoreceptors’ synaptic output: they receive vertical input (from photoreceptors in healthy retina, or directly from opsin activation in the diseased retina) and pass signals on to retinal RGCs, while interacting with horizontal and amacrine cells for lateral modulation. By conferring light sensitivity to BP cells, one can harness much of the downstream retinal circuitry including center-surround receptive fields, ON vs. OFF pathway separation, and temporal filtering, all of which are critical for natural vision.

Delivering channelrhodopsin to ON-BP cells restored distinct ON RGC light responses and, in some cases, led to OFF responses through network interactions, partially mimicking more natural signaling pathways (Lagali et al., 2008; Macé et al., 2015). The cell-tailored GPCR actuator Opto-mGluR6 (melanopsin–mGluR6 chimera) couples light to the native ON-BP Go/TRPM1 pathway, recovering retinal and cortical responses within the cone luminance range and with ON-BP cell–like latencies (van Wyk et al., 2015).

ON bipolar cell-selective expression is commonly achieved with GRM6 enhancers/mini-promoters or PCP2/L7 variants. In mice, combining GRM6 regulatory elements with optimized capsids yields broad ON-BP cell transduction e.g., AAV2-7m8 + 200-bp GRM6; AAV8.BP2 + 4 × GRM6 (Cronin et al., 2014; Macé et al., 2015). With GRM6-hRHO, rd1 mice detected 1:50 contrast steps (4-Hz flicker), an improvement in contrast sensitivity despite incomplete restoration (Cehajic-Kapetanovic et al., 2015). Comparative datasets indicate that targeting hOPN4 to ON-BP cells (e.g., L7-based constructs) produces shorter latencies and broader dynamic range than RGC-targeted or untargeted expression, consistent with upstream placement in the circuit (Gilhooley et al., 2022). Within the same study, untargeted (CBA) hOPN4 was evaluated alongside L7-targeted hOPN4, revealing briefer latencies and wider dynamic range with ON-BP cell targeting (Gilhooley et al., 2022). Where residual cone structure persists, ectopic rhodopsin can also drive the native phototransduction cascade, preserving signal polarity (Figure 3).

In addition to ON-BP cells, there may be cases where targeting OFF-BP or other interneurones could preserve more nuanced visual processing or enable certain contrast-detection pathways. While most current clinical-stage strategies focus on ON-BP cells (Cehajic-Kapetanovic et al., 2015; Lagali et al., 2008; van Wyk et al., 2015), future work might examine how dual-targeting or broader BP cell specificity affects vision restoration. Indeed, layering multiple constructs (for ON and OFF pathways) could theoretically recapitulate the retina’s center-surround and contrast enhancement features. However, the complexity of such an approach must be balanced against practical considerations for safety and manufacturability.

While targeting BP cells preserves more of the retina’s natural ON–OFF circuitry, the delivery route can strongly influence transduction efficiency. Intravitreal delivery is less invasive but historically had limited ON-BP cell access; engineered capsids such as AAV2-7m8 overcame this and restored both ON and OFF responses after intravitreal injection in mice (Macé et al., 2015). This level of expression is not achieved in NHP retinas more similar to that of humas (Dalkara et al., 2009). Subretinal routes can yield high local expression but treat a smaller area and have foveal surgical risks in advanced disease (Cronin et al., 2014; Macé et al., 2015). Furthermore, in advanced degenerations, BP cells sometimes undergo morphological changes (e.g., dendritic retraction) that may limit the therapeutic window for opsin expression (Marc et al., 2003; McClements et al., 2021). Future refinements in viral vectors and surgical techniques will be crucial to maximizing the functional integration of mammalian opsins in diseased retinas.

At the practical level, achieving robust and stable opsin expression in advanced inherited retinal disease requires specialized AAV capsids that transduce the degenerate retina and overcome anatomical barriers. Tyrosine-mutant AAV2 variants enhance intraretinal transduction (Gilhooley et al., 2022); in parallel, AAV2-7m8 (engineered heptamer insertion) and AAV8.BP2 (synthetic AAV8 variant) improve penetration to ON bipolars. Cellular specificity still derives primarily from the promoter (e.g., GRM6, L7). In rodent models, these capsid–promoter combinations achieve broad ON-BP cell expression by either intravitreal (AAV2-7m8) or subretinal (AAV8.BP2) delivery (Cronin et al., 2014; Gilhooley et al., 2022; Macé et al., 2015). Notably, ON-BP cell transduction with human rod opsin or channelrhodopsin variants has not shown adverse effects on retinal anatomy or ON-BP cell survival in mice (Wright et al., 2021). Ultimately, an ideal viral construct combines a robust capsid with an optimized promoter, balancing high expression levels against cellular specificity (Cronin et al., 2014; Gilhooley et al., 2022; Macé et al., 2015).

### 2.4 Safety and toxicology

One important concern in optogenetic therapy is to prevent retinal phototoxicity by ensuring stimulation within safe light exposure limits. The risk of damage is influenced by wavelength, irradiance intensity, and cumulative exposure duration with short-wavelength blue light (400–500 nm) being of particular concern. Its high-energy photons have the potential of inducing oxidative stress and photochemical damage to photoreceptors and the RPE (van Norren and Gorgels, 2011; Youssef et al., 2011). The historical primate damage threshold near 441–458 nm are 10–30 J/cm^2^, used by many as the onset-of-damage dose (van Norren and Gorgels, 2011). However, preclinical animal and *in vitro* work suggest damage at exposures up to approximately 20 times lower (Chakravarthy et al., 2024), therefore making some historical references overly permissive if used without geometry and duration adjustments. Accounting for spectrum, pupil and exposure geometry, if the true blue light threshold were indeed lower by a factor of 20, it would bring the revised estimate to around 1.1 J/cm^–2^. At this specific wavelength, when converted to photon flux, this corresponds to a cumulative dose of ∼2.46 × 10^18^ photons/cm^–2^.

Microbial-opsin stimulation in the retina typically requires retinal irradiances ≥10^15^ photons⋅cm^–2^⋅s^–1^- e.g., *in vivo* primate V1 activation at ∼9 × 10^15^ photons⋅cm^–2^⋅s^–1^, *ex vivo* NHP RGC responses around ∼2.3 × 10^15^ photons⋅cm^–2^⋅s^–1^, and clinical goggles delivering ∼4 × 10^14^–4 × 10^16^ photons⋅cm^–2^⋅s^–1^ at the retina (Chaffiol et al., 2022; Gauvain et al., 2021; Sahel et al., 2021). While this intensity is well below the phototoxic threshold in the short term, prolonged exposure could lead to damage. At 10^15^ photons⋅cm^–2^⋅s^–1^, the cumulative dose would reach the estimated phototoxic threshold in approximately 41 min, whereas at 10^14^ photons⋅cm^–2^⋅s^–1^, it would take about 6.8 h to reach the same threshold. This is in contrast with mammalian opsins which operate at 10^11^–10^12^ photons⋅cm^–2^⋅s^–1^, orders of magnitude lower than microbial opsins and well within known retinal safety limits. This strengthens the argument of a better safety profile of human, allowing for sustained activation without approaching toxic exposure levels.

A subset of recently characterized channelrhodopsins show improved photosensitivity compared to canonical tools- for example ChRmine, which exhibits large photocurrents and enhanced sensitivity linked to its pump-like channel architecture (Kishi et al., 2022). Likewise, the bacteriorhodopsin-like Guillardia theta cation channelrhodopsin (GtCCR4) displays higher light sensitivity than typical ChRs with minimal desensitization (Tanaka et al., 2024). We therefore qualify our generalization as tool-dependent; however, *in vivo* retinal implementations to date (e.g., ChrimsonR in the PIONEER study) still rely on device-assisted illumination in the ∼10^14^–10^16^ photons⋅cm^–2^⋅s^–1^ range (Sahel et al., 2021).

Red-shifted opsins, such as ReaChR and ChrimsonR, further enhance safety by shifting activation to longer wavelengths (∼500–600 nm), carrying less energy and consequently posing a lower phototoxic risk than blue light. The need for high-intensity illumination is further reduced by the ability of red light to penetrate biological tissues more efficiently and result in less scatter. ReaChR exhibits stronger membrane trafficking, expression and a more robust response above 600 nm than VChR1/C1V1 variants, addressing historical limits of red-shifted tools (Lin et al., 2013). ReaChR was shown to restore light responses in blind mice using intensities that appear lower than the safety threshold for the human retina (Sengupta et al., 2016). Similarly, ChrimsonR, with peak activation at ∼590 nm, maintains high sensitivity while avoiding the risks associated with high-energy short-wavelength exposure (Klapoetke et al., 2014). Retinal safety margins are more favorable at ∼590–595 nm (orange light), and experimental red-shifted stimulation in mouse, macaque and human retina was operated below blue-light hazard limits for those wavelengths (Sengupta et al., 2016). These advantages suggest that red-shifted opsins are an important tool for improving the safety profile of vision restoration therapies.

Unlike mammalian opsins, which engage intracellular G-protein signaling, microbial opsins function as ligand-gated cation channels, allowing uncontrolled influx of protons upon activation and directly modifying ionic homeostasis. The resulting prolonged membrane depolarization and intracellular acidification driven by H^+^ as a component of the overall cation current, has resulted in them effectively acting as “leaky pumps” (Feldbauer et al., 2009). The ensuing disruption in pH balance and altered cellular metabolism may trigger cytotoxic stress, especially in the context of more chronic expression. Excessive cationic load, such as from calcium influx, has been implicated in neuronal excitotoxicity and oxidative stress (Ermak and Davies, 2002). Furthermore, in the context of sustained depolarisation, the normal synaptic function of non-spiking cells such as retinal ON-BP cells could be particularly affected. While evidence from early studies does indeed indicate good tolerability of microbial opsins in the short term, the long-term consequences of persistent proton influx remain unknown.

Beyond channels and pumps, some microbial rhodopsins carry cytoplasmic enzymatic domains that couple light to cyclic-nucleotide modulation. Rhodopsin–guanylyl cyclases (Rh-GCs) such as BeCyclOp from *Blastocladiella emersonii* produce cGMP with fast kinetics and very high light/dark regulation (∼5,000-fold), enabling conductance changes via co-expressed CNG channels in cells and behavior in *C. elegans* (Gao et al., 2015). Related Rh-GCs (e.g., CaRhGC) show >1,000 light/dark activity ratios with signaling-state formation and decay on the tens-of-ms to subsecond scale, underscoring rapid control of cGMP (Scheib et al., 2018). Complementing cyclases, rhodopsin-phosphodiesterases (Rh-PDEs) enable light-driven cGMP degradation. Recent work identifies CfRhPDE1 with subsecond coupling and cGMP-selective hydrolysis suitable for bidirectional (Rh-GC + Rh-PDE) control of cGMP and downstream CNG currents (Liem et al., 2025). Earlier Rh-PDE variants (e.g., SrRh-PDE) also decrease cGMP and/or cAMP upon illumination, expanding the enzyme-opsin toolkit (Sugiura et al., 2020).

Multiple teams have reported no evidence of inflammation or toxicity from human opsin gene therapy over extended follow-up periods (Cehajic-Kapetanovic et al., 2015; De Silva et al., 2017; Lin et al., 2008). The immunogenicity of human opsins is expected to be low, given that rods, cones, and even intrinsically photosensitive retinal ganglion cells in normal humans express opsin-like proteins. Indeed, no substantial adverse immune responses were observed in rodent models treated with AAV-hOPN4 (De Silva et al., 2017; Gilhooley et al., 2022). Similarly, there was an absence of microglial activation or inflammatory markers in models treated with rhodopsin-based or MW-cone opsin-based vectors (Berry et al., 2019; Gaub et al., 2015).

Although mammalian opsins themselves appear relatively non-immunogenic (due to their endogenous nature) (Cehajic-Kapetanovic et al., 2015; De Silva et al., 2017; Lin et al., 2008), the viral delivery vehicles - most commonly AAVs - do carry an inherent risk of immune response. Several reports in rodent models have noted mild inflammatory signs when using high viral titres or repeated injections, even if no overt cytotoxicity is observed. They highlighted that vector dose, route of administration, and capsid serotype can influence the likelihood of T-cell or antibody-mediated reactions (Cronin et al., 2014; Macé et al., 2015). Reports show that intravitreal injections generally trigger stronger immune responses than subretinal delivery due to increased immune surveillance in the vitreous (Dalkara et al., 2009).

Retinal degeneration itself is associated with pro-inflammatory responses and electrical hyperactivity in RGCs. The non-steroidal anti-inflammatory drug meclofenamic acid can block gap junctions: 50 μM of meclofenamic acid significantly reduced spontaneous firing and increased signal-to-noise ratio across intensities (*ex vivo* MEA in rd1), without altering onset latency (Eleftheriou et al., 2017). The proposed mechanism is that noise suppression could indirectly dampen inflammation by reducing activity-dependent cytokine release. Given that its action is on postsynaptic connexin hemichannels it can mitigate degeneration-induced hyperexcitability without interfering with opsin biochemistry. Moreover, meclofenamic acid has an established systemic use and a relatively benign immune profile.

In addition, the pathological burst firing that emerges in RGCs after photoreceptor loss has been shown to be driven by excess retinoic acid (RA) signaling via RA nuclear receptors (RAR). In rd1 and rd10 mice, this hyperactivity can be silenced within minutes by a pan-RAR inverse agonist which unmasks light responses and improves behavioral vision (Telias et al., 2019). More recently, the aldehyde dehydrogenase (ALDH) inhibitor disulfiram, currently licensed for alcoholism, was repurposed to block RA synthesis. A single intraperitoneal dose reduced spontaneous RGC firing by 40% and doubled image detection performance in degenerate mice (Telias et al., 2022). These interventions reduce RA signaling (via RAR inverse agonism or ALDH inhibition) and quickly suppress degeneration-induced RGC hyperactivity, thereby unmasking light responses and improving behavioral vision in rd models; they are not expected to deplete 11-cis-retinal in the visual cycle. ChR2 binds the all-trans chromophore already abundant in retinal cells and it photo-isomerises back to the same all-trans form after each flash with no enzymatic recycling. RA blockage could be combined with channelrhodopsin-based therapies to suppress noise without sacrificing photosensitivity.

While these have typically been low-grade inflammatory responses in preclinical studies, they underline the need for careful dosing regimens and the possibility of pre-existing neutralizing antibodies in some patient populations. Ongoing Phase I/II clinical trials for optogenetic gene therapy now routinely include immunomodulatory regimens (e.g., brief oral corticosteroids) and detailed monitoring of intraocular inflammation to ensure that potential immune responses to the vector itself do not compromise the transplanted cells or long-term treatment efficacy.

### 2.5 Current trials

Given the microbial provenance of the optogenetic sensors under evaluation, each of the current intravitreal trials is first and foremost a safety study. Early-phase results are encouraging. One such trial is the PIONEER study, an open-label, low number trial where device-assisted functional signals have been reported. In this phase I/II study of ChrimsonR-tdTomato (GS030) in retinitis pigmentosa, a single low-dose intravitreal injection produced only mild, self-limited anterior chamber inflammation that was readily controlled with topical corticosteroids. The main complaint was transient photosensitivity, with no structural or systemic adverse events during 1-year follow-up (Sahel et al., 2021, 2022). However, vector doses used are very low and likely below threshold for efficacy.

Similar findings have been reported for the multi-characteristic-opsin programme: the phase 2 STARLIGHT trial (open-label, 48 weeks) enrolled six adults with Stargardt disease who received a single intravitreal dose of multi-characteristic opsin-010 (MCO-010) with short prophylactic oral or topical steroids. All participants had at least one ocular treatment emergent adverse effects (most commonly conjunctival hemorrhage, ocular hypertension, vitreous cells), but there were no serious adverse side effects at low doses used and inflammatory events were limited and steroid-responsive. Reported mean best corrected visual acuity change in treated eyes was +5.5 ETDRS letters at week 48 without a wearable magnifier; with a wearable low-vision magnifier, gains averaged +13.3 letters. A prespecified subgroup with macula-confined atrophy showed larger improvements (+12 letters without, +32 letters with magnifier) (Lam et al., 2025). Mean defect on perimetry improved by ∼2.6 dB at week 48, and Michigan Retinal Degeneration Questionnaire (MRDQ) patient-reported outcomes improved in reading and color/contrast domains (Lam et al., 2025). Fellow eyes also showed vision gains, underscoring potential learning or practice effects in this small open-label study (Lam et al., 2025). Open-label design, small numbers, and the use of a wearable magnifier for some endpoints limit inference and while overall safety and durability support continued development while, there is a clear need for randomized, controlled confirmation.

Furthermore, the phase 2b RESTORE trial in retinitis pigmentosa demonstrated a company-reported outcome of no serious ocular adverse events or drug-related systemic adverse events through 52 weeks of follow-up (Boyer et al., 2023). Finally, the phase I/II trial of the channelrhodopsin construct RST-001 likewise documented only low-grade anterior and posterior segment inflammation that resolved with topical or periocular steroids and reported no procedure- or vector-related complications (AbbViNational Library of Medicine (US)e, 2024). Notably, two of these vectors (GS030 and the BS01) incorporate fluorescent protein fusion tags; although long-term data are still pending, no tag-related immunogenicity has yet emerged. Collectively, these results suggest that the modest vector doses now being deployed sit below the threshold for clinically significant ocular toxicity, while efficacy signals such as partial visual recovery in PIONEER, indicate that higher, yet still safe, dose levels may be required to reach full therapeutic potential (Sahel et al., 2021, 2022).

Nonetheless, large animal and clinical studies must continue to assess long-term cytotoxicity, potential off-target expression, and any functional deficits to the inner retina. Pre-clinical studies are promising with regards to reporting no excitotoxicity or functional impairments in the case of both a 9-months period following intravitreal injection (Wright et al., 2021) and a 13-months period following subretinal delivery (De Silva et al., 2017), however the questions remain as to whether this is translatable to more extensive follow-up or clinical trials. As these therapies progress to human trials, comprehensive toxicology and immunological assays remain a priority, which mandates close examination of repeated-dose paradigms and multi-year evaluations to fully assess off-target risks, immunogenicity, or functional drift.

### 2.6 Clinical context and patient selection

Human-opsin optogenetics is most likely to help patients with advanced photoreceptor loss but preserved inner retinal circuitry, a configuration common in late-stage IRDs including macular and cone dystrophies, and in atrophic areas of geographic atrophy related to age-related macular degeneration (AMD). Histology and OCT studies show that, even late, the inner retina often persists (though remodeled), providing a receptive substrate for ectopic opsins. In a recent end-stage IRD cohort, 46.3% of eyes still had discernible inner layers and/or thickened inner nuclear layer (INL) on SD-OCT (mean foveal INL ≈29 ± 11 μm), supporting candidacy for inner-retina targeting (Jones et al., 2012; Ng et al., 2025; Strettoi, 2015). A very few, if any patients in our group had discernible photoreceptor inner segments suitable for optogenetic targeting. Proposed patient selection criteria for functional rescue by human opsins: (i) SD-OCT evidence of preserved INL and ganglion cell/ inner plexiform layer (GC/IPL) at the fovea/parafovea (qualitative continuity and lamination; INL commonly ∼20–30 μm in late-stage cohorts), (ii) SD-OCT absence of profound inner-retinal atrophy/lamination loss, (iii) intact optic nerve and media (Ng et al., 2025). For atrophic AMD, similar logic applies where inner retina is spared within or adjacent to atrophy (Borchert et al., 2024).

Clinical endpoints that map well from preclinical to clinic include full-field stimulus threshold (FST) for global light sensitivity, fundus-tracked microperimetry for mesopic/photopic function, task-based functional vision (object detection/mobility under calibrated lux), visual acuity tests adapted for ultra-low vision (electronic reading tests, Landolt *E*-test) and patient reported outcomes (Thirunavukarasu et al., 2025). Post treatment there is likely to be a period of visual rehabilitation as seen previously with electronic retinal implants (Cehajic Kapetanovic et al., 2020).

## 3 Discussion

Despite considerable progress, clinical translation in optogenetics is constrained by current therapies being solely based on microbial opsins. The studies have been ongoing for a while now, without major breakthroughs or approved therapies. And indeed, we desperately need new treatments for late stage degenerations, as current gene therapy and gene editing approaches may not suitable for this group of patients (Cehajic Kapetanovic et al., 2019; Cehajic-Kapetanovic et al., 2020, 2024; Kantor et al., 2021; Kiraly et al., 2023; Lam et al., 2024; MacDonald et al., 2020; Martinez-Fernandez de la Camara et al., 2022). First-in-human trials with microbial opsins have reported limited vision restoration, with challenges due to high light requirements and vector-related intraocular inflammation (AbbViNational Library of Medicine (US)e, 2024; Boyer et al., 2023; Lam et al., 2025; Sahel et al., 2021). Patients with impaired blink and pupillary reflex may accrue chronic, cumulative light exposure whose safe upper limits are not fully defined, keeping phototoxicity squarely on the agenda. Additionally, microbial proteins packaged at high AAV titres and delivered to ectopic membranes can provoke adaptive response resulting in undesirable immune reactivity. These considerations create a compelling rationale to prioritize alternative clinical optogenetic approaches, including human based opsins, supported by encouraging pre-clinical performances. These first-in-human trials will be crucial in determining the quantity and quality of restored responses backed up by very promising pre-clinical data.

Future efforts will likely focus on improving vector safety and delivery, the light sensitivity and kinetics of ectopically expressed opsins, refining capsid/promoter combinations, and integrating these approaches with remaining photoreceptor functions in patients at different stages of advanced retinal degeneration (Gilhooley et al., 2022). In parallel, more thorough behavioral and psychophysical assessments in large-animal models and human patients will clarify how well light adaptation and contrast sensitivity are restored in real-world conditions, so that meaningful vision such as face recognition is restored.

One area needing further refinement is durable chromophore regeneration and photostability. Human opsins must bind 11-cis-retinal to remain photosensitive, yet in end-stage degeneration, the canonical visual cycle may be compromised. When ectopically expressed in surviving retinal neurones, these pigments risk stalling in bleached, all-trans conformation, precipitating a progressive decline in light responses. Empirical approaches, such as systemic 9-cis-retinal supplementation, co-expression of retinal isomerases or the design of intrinsically bistable pigment chimeras have yielded promising short-term restoration in murine models (Katada et al., 2023; Lucas et al., 2014; Mure et al., 2016), but longer-term stability and scalability are yet to be demonstrated. Quantitative assays of chromophore turnover in diseased human tissue and long-term studies confirming response durability are indispensable prerequisites for clinical translation.

For restoration of useful vision, simple delivery of genetic material through a vector is not enough. The ectopic proteins must couple to an intact G-protein cascade, drive downstream ion-channel modulation with sub-second kinetics and manage to do so across the highly remodeled landscape of end-stage regeneration (Jones et al., 2003; Marc et al., 2003). Several studies already hint at cracks in this mechanistic chain. In advanced disease, the availability and functionality of key cascade components such as transducin, PDE6, c-GMP gated channel for photoreceptor target or TRPM1 channels remain uncertain and this might mean that opsin responses decay below the temporal bandwidth required for high-level vision. A more precise molecular mapping of downstream signaling reserves in degenerate mouse and human donor retina may help answering these questions. Quantitative proteomics and single-cell transcriptomics of late-stage retinas could establish which G-protein subunits, effector enzymes and channels persist and at what levels, before committing trials to a given cell target or opsin design.

Bio-engineered opsins can also help achieve faster, tuneable kinetics. Chimeric constructs already demonstrate that domain swapping can shorten response latencies while retaining amplification (Katada et al., 2023; van Wyk et al., 2015), but this has not yet been benchmarked beyond rodent eyes. Future models must show scalability to primate retinas.

While current strategies have managed to improve contrast detection and simple navigation, wild-types acuity and flicker discrimination are yet to be achieved (Berry et al., 2019; Cehajic-Kapetanovic et al., 2015). In order to restore useful vision, high frequency modulation must be attained across both mesopic and photopic conditions.

Early human data (PIONEER/STARLIGHT/RESTORE) indicate acceptable safety at modest doses with emerging efficacy signals (AbbViNational Library of Medicine (US)e, 2024; Boyer et al., 2023; Lam et al., 2025; Sahel et al., 2021, 2022), strengthening the case for Phase 1/2 studies of human-opsin vectors with prospectively defined light-dose budgets and safety as well as function-orientated endpoints (contrast sensitivity, mesopic mobility, temporal contrast sensitivity function). As such, successful deployment of human-opsin gene therapy hinges on durable chromophore recycling, engagement of a sufficiently rapid and intact intracellular cascade, and adoption of evidence-based safety frameworks acceptable to regulators. Progress across these axes is interdependent; advancing them in concert is the fastest path from experimental promise to clinically meaningful restoration of light sensitivity.
